# Assessing the Predictive Accuracy of Open Injury Scores in Determining the Salvageability of Type IIIB Tibial Fractures

**DOI:** 10.7759/cureus.102965

**Published:** 2026-02-04

**Authors:** Utkarsh Bansal, Devarshi Rastogi, Shatakshi Pant, Mayank Mahendra, Brij Mohan Patel

**Affiliations:** 1 Orthopaedic Surgery, King George's Medical University, Lucknow, IND

**Keywords:** ghoiss, limb salvage, lsi, mess, type iiib open tibial fracture

## Abstract

Severe open tibial fractures pose a major challenge in orthopaedic trauma care. The decision to salvage or amputate a limb requires objective assessment tools. The Mangled Extremity Severity Score (MESS), Limb Salvage Index (LSI), and Ganga Hospital Open Injury Severity Score (GHOISS) are widely used, but their accuracy in Type IIIB open tibial fractures remains under evaluation. This study aimed to validate these scoring systems and determine the most reliable predictor of limb salvageability. A prospective observational study was conducted on 307 patients with Type IIIB open tibial fractures at the Department of Orthopaedic Surgery of a tertiary care centre in North India. Patients were assessed using MESS, LSI, and GHOISS after the first surgical debridement. Limb salvageability was defined as limb retention at six months. Statistical analysis included chi-square tests and receiver operating characteristic (ROC) curve analysis. The majority of patients were male (85%), with a mean age of 28.67 ± 9.28 years. Road traffic accidents (72.3%) were the most common cause of injury. Limb salvage was achieved in 93.5% of cases, while 6.5% underwent amputation, particularly in cases within the grey zone (scores 15-16). Higher MESS, LSI, and GHOISS scores were significantly associated with amputation (p < 0.001). ROC analysis showed that GHOISS (AUC = 0.997) had superior predictive accuracy compared with MESS (AUC = 0.958) and LSI (AUC = 0.953). GHOISS demonstrated higher accuracy and specificity in predicting limb salvageability within this cohort, suggesting that it may be a useful tool to support clinical decision-making in Type IIIB open tibial fractures.

## Introduction

Open tibial fractures, particularly those classified as Gustilo-Anderson Type IIIB, present a significant challenge to orthopaedic surgeons due to the complexity of managing both bony and soft tissue injuries [1,2]. These fractures are characterised by extensive soft tissue damage, periosteal stripping, and significant contamination, often requiring a multidisciplinary approach involving orthopaedic, plastic, and vascular surgeons to optimise outcomes [1]. The primary goals in managing Type IIIB open tibial fractures are to prevent infection, achieve bony union, and restore limb function [3]. However, the decision between limb salvage and amputation remains a critical and often difficult one, influenced by factors such as the severity of the injury, the presence of vascular compromise, and the overall general condition of the patient [4].

The management of open tibial fractures has evolved significantly over time. Historically, immediate amputation was often the preferred approach for severe open fractures [4]. However, with advancements in surgical techniques, antibiotic therapy, and microsurgical reconstruction, limb salvage has become an increasingly viable option [4,5]. Despite these advancements, there remains a lack of consensus on the optimal treatment strategy, prompting the development of various prognostic scoring systems aimed at aiding the decision-making process [4].

Several scoring systems have been developed to assess the severity of open fractures and predict the likelihood of successful limb salvage. These include the Mangled Extremity Severity Score (MESS), the Limb Salvage Index (LSI) [4], and the Ganga Hospital Open Injury Severity Score (GHOISS) [2]. GHOISS is specifically designed to evaluate the severity of injuries to the covering tissues, bones, and functional tissues, and it considers comorbid conditions that may influence management and prognosis [2]. These scoring systems aim to provide a more objective assessment of the injury and help guide treatment decisions, although their reliability and predictive value can vary.

## Materials and methods

This prospective observational study was conducted at the Department of Orthopaedic Surgery of a tertiary care centre to evaluate and compare the predictive accuracy of three injury scoring systems, the GHOISS, MESS, and LSI, in determining limb salvageability in patients presenting with Gustilo-Anderson Type IIIB open tibial fractures. Ethical clearance for the study was obtained from the Institutional Ethics Committee.

The study included 307 patients aged 15-65 years who presented within 24 hours with Gustilo-Anderson Type IIIB open tibial fractures. Patients with Type IIIC fractures, near-total traumatic amputations, irreparable soft tissue injuries, and upper limb trauma were excluded to maintain a homogeneous study population. The sample size was calculated based on outcomes reported by Rajshekharan et al. (2006) [2], in which the area under the curve (AUC) for MESS was 0.960 ± 0.028 and for GHOISS was 0.964 ± 0.025. A two-sided Z-test was applied with α = 0.05 and power = 80% (β = 0.20).

**Figure 1:**
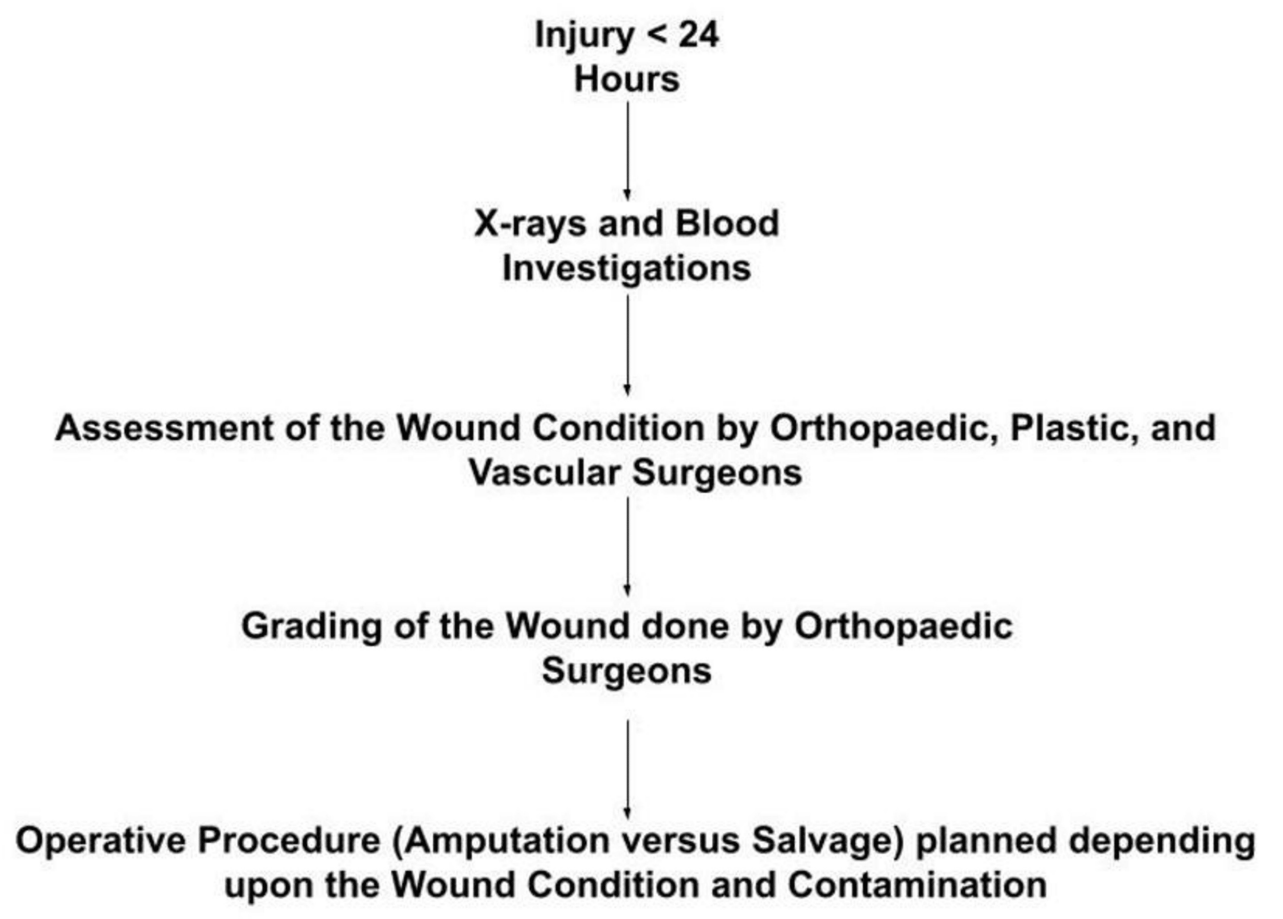
Early triage and surgical decision protocol for limb injuries (<24 hours). Flowchart depicting the standardised clinical pathway followed for patients presenting with lower limb trauma. Patients presenting within 24 hours of injury underwent radiographic and blood investigations, followed by multidisciplinary wound assessment by orthopaedic, plastic, and vascular surgeons. Wound grading was performed by orthopaedic surgeons, after which the decision for limb salvage or amputation was made based on wound severity and contamination status.

Each patient underwent a comprehensive clinical and radiological evaluation upon admission. Injury severity scores were calculated using the GHOISS, MESS, and LSI scoring systems after the first surgical debridement. These scores were assigned by an independent observer who was blinded to the eventual outcome to minimize bias. The decision to proceed with limb salvage or amputation was made independently by the treating orthopaedic surgeons, based solely on clinical and intraoperative judgment (Figure 1).

**Figure 2:**
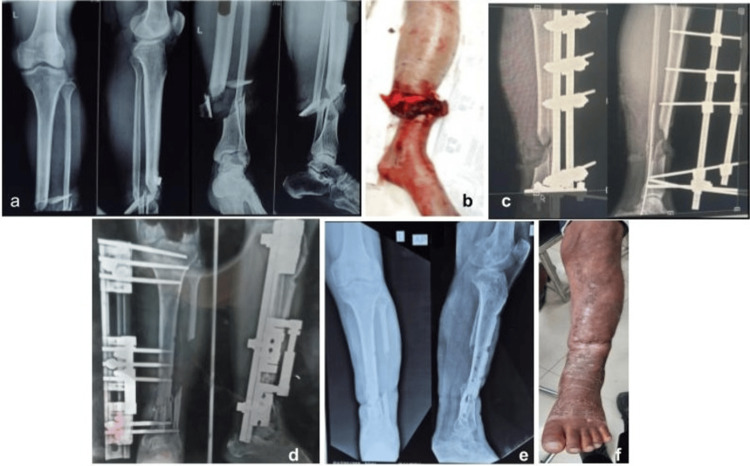
Radiological and clinical progression following management of left leg injury. A 52-year-old male patient managed with wound debridement and regular dressing along with external fixator application for a left leg injury. (a) Pre-operative anteroposterior and lateral radiographs showing the initial fracture pattern. (b) Pre-operative clinical photograph demonstrating the extent of soft tissue injury. (c) Immediate post-operative radiograph following external fixator application. (d) Three-month follow-up radiograph showing progressive fracture healing and maintained alignment. (e) Six-month follow-up radiograph demonstrating satisfactory bone union. (f) Six-month follow-up clinical photograph showing healed soft tissue and functional limb preservation.

**Figure 3:**
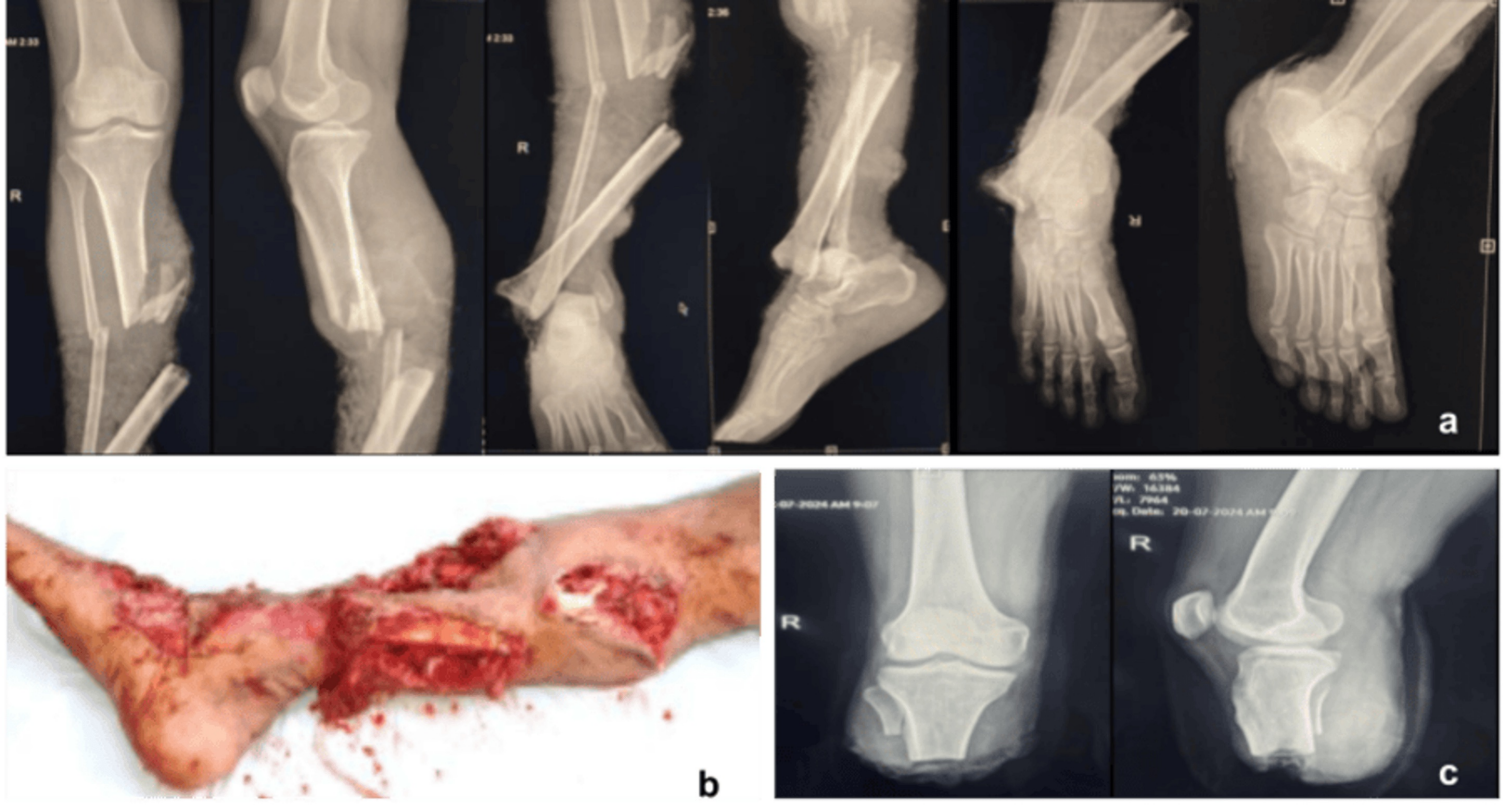
Radiological and clinical findings in a patient undergoing below-knee amputation. A 30-year-old male patient managed with wound debridement and dressing followed by right-sided below-knee amputation. (a) Pre-operative anteroposterior and lateral radiographs demonstrating the extent of bony injury. (b) Pre-operative clinical photograph showing severe soft tissue damage. (c) Post-operative radiograph confirming completion of below-knee amputation with satisfactory stump formation.

The primary outcome was the final limb status at six months post-injury, categorized as either salvaged (Figure 2) or amputated (Figure 3). Follow-up assessments were conducted at six weeks, three months, and six months to track progress and assess the outcome. Data were compiled and statistically analyzed using IBM SPSS Statistics for Windows, Version 24 (Released 2016; IBM Corp., Armonk, New York). Receiver operating characteristic (ROC) curve analysis was performed, and statistical significance of the area under the curve (AUC) was tested against the null hypothesis of AUC = 0.5 using a Z-test. A p-value of <0.05 was considered significant.

## Results

A total of 307 patients in the age group of 16-65 years with Gustilo-Anderson Type III B open tibial fractures were enrolled. The mean age of the enrolled patients was 28.67 ± 9.28, with the majority of male patients aged between 20 and 40 years. Road traffic accidents were the most common mode of injury (72.3%), and most patients presented within six hours of trauma (77.5%). Mixed-mechanism injuries were predominant (92.2%), and 36.2% had associated injuries, most commonly facial (21.2%) and chest (14.7%) (Table 1). At six months, 287 (93.5%) limbs were salvaged, while 14 limbs (4.56%) and six limbs (1.95%) underwent primary and secondary amputations, respectively. 

**Table 1:** Demographic details of the enrolled patients.

Characteristics	No. of Patients	Percentage
			Age group (years)		
			≤20	19	6.2
21-30	207	67.4
31-40	43	14
41-50	26	8.5
≥51	12	3.9
Gender		
Female	46	15
Male	261	85
Mode of injury		
Fall of heavy object	2	0.7
Machine injury	19	6.2
Physical assault	63	20.5
Road traffic accident	222	72.3
Train accident	1	0.3
Mechanism of injury		
Blunt injury	22	7.2
Mixed	283	92.2
Penetrating	2	0.7
Associated injury		
None	183	63.8
Chest injury	45	14.7
Head injury	20	6.5
Facial injury	65	21.2
Time elapsed from injury (hours)		
<6 hours	238	77.5
≥6 and <12 hours	9	2.9
≥12 hours and <24 hours	60	19.6

**Figure 4:**
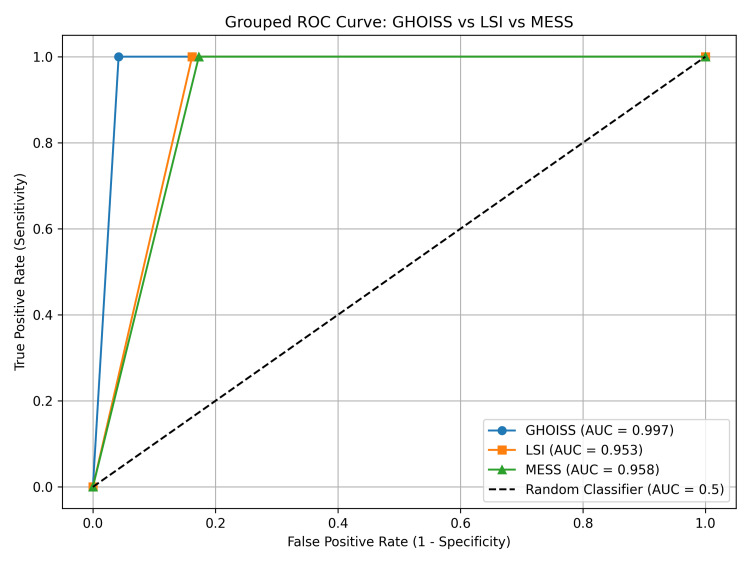
Grouped ROC curve for GHOISS, MESS, and LSI. Receiver operating characteristic (ROC) curves comparing the diagnostic performance of the Ganga Hospital Open Injury Severity Score (GHOISS), Limb Salvage Index (LSI), and Mangled Extremity Severity Score (MESS) in predicting limb salvage outcomes. The area under the curve (AUC) values demonstrate excellent discrimination for GHOISS (AUC = 0.997), followed by MESS (AUC = 0.958) and LSI (AUC = 0.953). The dashed diagonal line represents a random classifier (AUC = 0.5).

The diagnostic accuracy of the three scoring systems, GHOISS, MESS, and LSI, was compared using receiver operating characteristic (ROC) curve analysis. Among them, GHOISS showed the highest predictive performance with an AUC of 0.997, followed by MESS (AUC = 0.958) and LSI (AUC = 0.953) (Figure 1). At optimal cutoff values, determined by Youden’s Index, GHOISS (≥15.5) yielded 100% sensitivity and 95.8% specificity, while MESS (≥5.5) and LSI (≥3.5) achieved 100% sensitivity with specificities of 82.7% and 83.8%, respectively (Table 2 and Figure 4).

**Table 2:** Diagnostic performance of injury scoring systems. The z-test was used for calculation. *Significant p-value. GHOISS: Ganga Hospital Open Injury Severity Score; MESS: Mangled Extremity Severity Score; LSI: Limb Salvage Index.

Scoring System	Cut-off Score	Sensitivity (%)	Specificity (%)	AUC	p-Value
GHOISS	≥15.5	100	95.8	0.997	<0.001*
MESS	≥5.5	100	82.7	0.958	<0.001*
LSI	≥3.5	100	83.8	0.953	<0.001*

A statistically significant association was observed between higher injury scores and eventual limb amputation in all three scoring systems (p < 0.001). GHOISS uniquely identified a diagnostic "grey zone" between scores 15 and 16, offering a clinical buffer for individualised decision-making. In contrast, MESS and LSI lacked this stratification ability, tending to overpredict amputation in borderline salvageable cases. These findings affirm the superiority of GHOISS in accurately guiding limb salvage decisions in severe open tibial fractures.

In the ROC analysis of the three scoring systems for predicting limb salvageability, the GHOISS curve lies closest to the upper left corner, indicating the highest overall accuracy.

## Discussion

Type III B open tibial fractures are among the most complex orthopaedic injuries, often requiring difficult decisions regarding limb salvage versus amputation. The Gustilo-Anderson classification remains the most widely used system for open fractures and serves as a common language among clinicians and researchers. Its simplicity and familiarity contribute to its continued use; however, it has notable limitations. It compresses a wide spectrum of injury patterns into only a few categories, leading to poor interobserver reliability and inconsistent application. Moreover, it does not adequately account for evolving tissue viability, comorbidities, or the deeper extent of soft tissue damage, which are crucial in guiding treatment and predicting outcomes. These limitations underscore the need for more comprehensive and objective scoring systems in complex injuries like Type IIIB fractures [6]. This study aimed to compare the predictive performance of three commonly used injury scoring systems, GHOISS, MESS, and LSI, in determining salvageability.

Among the 307 patients included, 93.5% underwent successful limb salvage and 6.5% required amputation. The decision was taken by consensus of orthopaedic, plastic, and vascular teams based on wound condition, neurovascular status, contamination, and patient condition. Most patients presented within six hours of injury, and the mean GHOISS, MESS, and LSI scores were 13.1, 4.67, and 3.39, respectively, suggesting overall moderate injury severity in this cohort.

The MESS score, despite its widespread use, demonstrated limitations in this study. With an AUC of 0.958 and a cutoff of ≥5.5, it showed 100% sensitivity but lower specificity (82.7%). This confirms earlier criticisms of MESS, particularly its emphasis on vascular injury and shock, which may not be present in all Type IIIB injuries. Furthermore, MESS offers limited insight into soft tissue and bone damage, which are crucial for prognosis. The LSI also demonstrated good statistical performance (AUC 0.953; sensitivity 100%; specificity 83.8% at a cutoff ≥3.5). However, similar to MESS, it places greater emphasis on vascular parameters and provides relatively limited detail regarding the extent of musculoskeletal injury. Although both MESS and LSI showed high diagnostic accuracy, their utility in guiding reconstructive planning or stratifying borderline cases may be limited, particularly in injuries where soft tissue damage predominates.

To address the dilemma of limb salvage versus amputation in Gustilo type III patients, Rajasekaran and colleagues established the Ganga Hospital Open Injury Severity Score (GHOISS) in 1994 and validated it in 2006 [7-10]. The GHOISS has been shown in earlier research to be useful in predicting whether limb preservation or amputation is required for Gustilo type IIIB and IIIA injuries [11]. Additionally, the GHOISS has demonstrated equivalent sensitivity and specificity for limb amputation or salvage in children and adults [9, 12]. In particular, the best sensitivity and specificity for limb loss have been linked to a GHOISS of 17 or higher, whereas a score of 14 or lower has been linked to successful salvage [7, 9, 10].

In contrast to MESS and LSI, the GHOISS emerged as the most reliable tool in this study. It achieved near-perfect diagnostic accuracy with an AUC of 0.997 and 100% sensitivity and 95.8% specificity at a cutoff ≥15.5. GHOISS uniquely accounts for skin, bone, muscle involvement, and comorbid factors, offering a tissue-specific approach. This allows it not only to predict amputation risk more accurately but also to guide reconstruction plans. For instance, skin scores >3 indicated the need for flaps, and bone scores ≥4 predicted complex reconstructions such as bone grafting or transport. Okuku et al. (2025) reported an AUC of 0.923 for GHOISS in a Ugandan cohort, with optimal sensitivity and specificity at a cutoff of 13 [10]. Ndlovu et al. (2023) found a pooled sensitivity of 93.4% and specificity of 95% at a cutoff of 14 [13]. Gupta et al. (2020), in a high-volume Indian centre, also supported the superiority of GHOISS, particularly for its inclusion of a "grey zone" (scores 15-16) that allows for individualised clinical judgement [14].

The inclusion of a grey zone in GHOISS is crucial. It acknowledges variability in injury severity and institutional capabilities, offering a practical middle path where final decisions can be made by combining objective scores with surgeon expertise. In this study, no amputations occurred for scores ≤15, while all amputations occurred at scores ≥16, reinforcing the utility of the proposed cutoff.

Compared with prior studies, the lower amputation rate in this study (6.5%) may reflect earlier presentation, moderately severe injuries, and timely intervention. For instance, Gupta et al. (2020) reported an amputation rate of 12.6%, and Okuku et al. (2025) reported a rate of 12.2%, attributed to a resource-limited setting. Differences in patient selection, availability of reconstruction services, and institutional protocols likely contributed to this variation [10,14].

While MESS and LSI remain valuable, particularly in cases with vascular involvement, they provide comparatively limited information regarding comprehensive injury assessment and surgical planning. In this study, GHOISS demonstrated better discriminatory performance within the cohort. However, given the single-centre design and low amputation rate, these findings should be interpreted cautiously. GHOISS may be a useful adjunct in clinical decision-making, especially in resource-limited settings, but broader external validation is required before recommending its routine use in clinical practice.

However, limitations exist. This study represents an internal validation conducted at a single tertiary care centre, and the cohort had relatively low mean injury scores, which may limit generalisability to more severe injuries, particularly Type IIIC fractures. Although scoring was performed by a blinded independent observer, a degree of subjective variation in score interpretation among surgeons cannot be entirely excluded, which may have influenced assessment. Future multicentre studies with broader injury spectra and external validation are required to strengthen these findings.

## Conclusions

This study evaluated and compared the performance of three widely used open injury scoring systems, GHOISS, MESS, and LSI, in predicting limb salvageability in Type IIIB open tibial fractures. Among 307 patients, 93.5% achieved limb salvage, while 6.5% required amputation. All three scores demonstrated good diagnostic accuracy; however, the Ganga Hospital Open Injury Severity Score (GHOISS) emerged as the most reliable tool. At a cutoff ≥15.5, GHOISS showed 100% sensitivity and 95.8% specificity, outperforming both MESS and LSI. While MESS and LSI are useful in evaluating vascular injuries, they fall short in assessing the complex soft tissue and bone damage characteristic of Type IIIB injuries. GHOISS, in contrast, provides a tissue-specific and comprehensive evaluation, allowing for nuanced clinical decision-making. Its inclusion of a "grey zone" (scores 15-16) adds practical flexibility in borderline cases.

In our opinion, these findings underscore the superiority of GHOISS in both diagnostic accuracy and clinical utility. Given its ability to guide surgical planning and predict outcomes more precisely, GHOISS should be adopted as the preferred scoring system for managing Type IIIB open tibial fractures. Its routine use can enhance early decision-making, improve patient outcomes, and optimise resource utilisation, especially in high-volume trauma centres.
